# Hypercalcemia during pregnancy: management and outcomes for mother and child

**DOI:** 10.1007/s12020-021-02615-2

**Published:** 2021-02-05

**Authors:** Natasha M. Appelman-Dijkstra, Diana- Alexandra Ertl, M. C. Zillikens, Lars Rjenmark, Elizabeth M. Winter

**Affiliations:** 1 Department of Internal Medicine, Division of Endocrinology, Center for Bone Quality, Leiden University Medical Center, Leiden, the Netherlands; 2 Department for Pediatric Pulmonology, Allergology and Endocrinology, Comprehensive Center for Pediatrics, Medical University of Vienna, Vienna, Austria; 3 Center of Expertise for Rare Disorders of Bone, Growth and Mineralization, Vienna Bone and Growth Center, Vienna, Austria; 4 Department of Internal Medicine, Erasmus MC Bone Center, Erasmus University Medical Center, Rotterdam, the Netherlands; 5 Department of Clinical Medicine; Department of Endocrinology and Internal Medicine, Aarhus University Hospital, Aarhus, Denmark

**Keywords:** Pregnancy, Hypercalcemia, Familial hypocalciuric hypercalcemia, Parathyromatosis, Primary hyperparathyroidism

## Abstract

Diagnosing and treating hypercalcemia during pregnancy can be challenging due to both the physiological changes in calcium homeostasis and the underlying cause for the hypercalcemia. During pregnancy and lactation there is increased mobilization of calcium in the mother to meet the fetus’ calcium requirements. Here we discuss the diagnostic challenges, management, and patient perspective of hypercalcemia during pregnancy in two particular cases and in other rare conditions causing hypercalcemia.

## Introduction

Hypercalcemia in pregnancy is rare and in >90% of cases caused by a newly diagnosed primary hyperparathyroidism (PHPT). Hypercalcemia during pregnancy due to other disorders of calcium metabolism is even more rare and literature regarding the maternal management and fetal outcomes is lacking. Diagnosing hypercalcemia is challenging during pregnancy, as symptoms such as fatigue or nausea mimic those in early pregnancy. However, longstanding hypercalcemia might induce nephrolithiasis, pancreatitis, and preeclampsia in the mother [1]. Therefore, the occurrence of these diseases during pregnancy should push the physicians to screen for hypercalcemia. In the fetus, maternal hypercalcemia can result in fetal growth restriction [2]. Following birth, further complications may arise such as severe neonatal hypocalcemia, tetany, and even death due to fetal hypoparathyroidism [3, 4]. In rare calcium-related disorders, the diagnosis is often known before the pregnancy and patients and partners can be counseled by the treating endocrinologist, obstetrician, pediatrician, and geneticist when required, taking into account the possible physical and mental maternal adverse effects, but also the eventual consequences for the newborn. Here we would like to discuss the diagnostic challenges and management of hypercalcemia during pregnancy in two particular cases as example of rare conditions causing hypercalcemia complicating pregnancy.

### Case 1

A 34-year-old woman was referred with severe hypercalcemia and a pregnancy wish. At the age of 24 she was diagnosed with PHPT, without underlying genetic causes. During parathyroidectomy (PTX) there was spill of the adenoma, histologically confirmed, which led to chronic hypercalcemia for which she underwent several re-operations in various hospitals including a thyroidectomy in 2 tempi with resection of surrounding fat-tissue and a bilateral modified lymph node resection. However, she remained dependent on cinacalcet and bisphosphonates in various dosages. At referral, albumin-corrected serum calcium was 2.89 mmol/L (ref 2.15–2.55 mmol/L) with PTH 10.7 (ref 1–8 pmol/L) while on cinacalcet 60 mg/day, and 3.16 mmol/L with PTH of 29 without cinacalcet. She and her partner were counselled by a team consisting of gynecologists, pediatricians, and endocrinologists on several aspects of the disease and potential consequences for a pregnancy. Prophylactic surgery was not possible at that time. Bisphosphonates were stopped and she became pregnant after 12 months. Cinacalcet was stopped and a fluid intake of 4 L was advised. However, she was admitted with a symptomatic hypercalcemia, 3.4 mmol/L, within 2 weeks. Hyperhydration and cinacalcet, up to 90 mg/day, were started in combination with furosemide 40 mg and acetylsalicylic acid 80 mg daily as preeclampsia prophylaxis. She suffered from severe hyperemesis gravidarum and due to persistent hypercalcemia, she received saline infusions of 5 L/day in a homecare setting with a peripheral intravasal central catheter (PICC). During follow up the PICC caused deep vein thrombosis for which nadroparin injections were started but with this treatment serum calcium remained stable around 2.7–2.9 mmol/L. At 38 weeks of gestation she developed hypertension with a rapidly developing HELLP syndrome and an emergency cesarean section was performed. A healthy baby boy, weight 3305 g, was delivered; he only received active vitamin D supplementation for a few days and two years after delivery; no calcium disturbances or skeletal abnormalities have been observed. The mother did not breastfeed. After delivery, the mother experienced a post-traumatic stress syndrome (PTSS) with depression for which she started Eye Movement Desensitization and Reprocessing (EMDR). The depression and PTSS was related to the latter part of the pregnancy and delivery, but most likely exacerbated due to a postpartum rise in calcium to 3.3 mmol/L. Surgery was successfully performed after which her mental complaints improved.

### Case 2

A 38-year-old female was diagnosed with PHPT at a non-university hospital, based on a modest hypercalcemia, and elevated PTH of 9.2 pmol/L and high to normal urinary calcium excretion of 8.5 g/L and calcium-to-creatinine clearance above 0.02. Despite her young age, it was decided not to perform PTX because of an increased risk for venous thromboembolism and obesity. Five years later, she had an unexpected pregnancy and was referred for PTX in the second trimester. During that time serum calcium had increased from 2.63 mmol/L to 2.83 mmol/L with a non-suppressed PTH of 3.8 pmol/L. The patient and her partner were counseled by a team consisting of endocrinologists, anesthesiologists, endocrine surgeons, pediatricians, and obstetricians. Imaging studies did not reveal a clear-cut adenoma and considering the progressive rise in calcium to 2.93 mmol/L, urinary calcium excretion of 12 g/L in 24 h and proceeding into the end of the second trimester an elective neck exploration was performed at gestational age of 24 + 2 weeks, showing four gland hyperplasia. During surgery, it was decided to remove only the two right-sided glands in order not to risk hypoparathyroidism. After surgery hypercalcemia persisted with calcium levels between 2.60 mmol/L and 2.65 mmol/L and PTH between 2.1 pmol/L and 3.6 pmol/L. Considering the marked hyperplasia the patient was discussed in the clinical patient management system (CPMS) panel of main thematic group two, calcium and phosphate disorders of the European Reference Network for rare endocrine diseases (ENDO-ERN). The advice of this panel was to perform genetic testing for familial hypocalciuric calciuria (FHH), which came back positive for a mutation in the CASR-gen, c.653A>G p.(Tyr218Cys), establishing a diagnosis of FHH type one. At 39 + 3 weeks she delivered a healthy baby girl of 4150 g without any calcium disturbances. Post-partum the calcium levels of the mother remained at 2.6 mmol and PTH rose again to 10.3 pmol/L, which has been stable since.

#### Patient perspective

Patient one had an active pregnancy wish for 5 years, with a negative counselling in several centers. At the Leiden University Medical Center (LUMC) it was again made clear that this would not be without risks for mother and child but if desired we would do our best to monitor the pregnancy as good as possible, which was much appreciated as this was not discussed before. Although she was well prepared and extensively counseled, the intensity of the pregnancy was overwhelming. Both the high calcium levels, frequent investigations and the continuous hyperhydration were extremely demanding and resulted, together with the further rise in calcium, in a pre- and post-natal depression for which EMDR and psychotherapy were needed. Looking back, she would advise other patients to realize that it will be extremely tough, but absolutely worth it. The fact that caretakers were so involved in her well-being and their clear communication was helpful and appreciated. The health care system structure with various doctors during hospital admission remains unfortunate from her point of view, which was in contrast to the outpatient clinic care with one regular healthcare provider.

Despite living with, a supposed, PHPT for many years, patient 2 had never been counseled about PHPT in terms of treatment and follow up. In the initial phase of her referral to LUMC this was done rapidly as time was ticking for a safe window of surgery as the second trimester is the preferred trimester for surgery for maternal and fetal reasons. In addition, due to her increased risk for venous thromboembolism and obesity, emergency surgery in case of severe hypercalcemia was considered to be a high risk for the mother. The patient found the amount of information overwhelming. However, it was appreciated as it made the patient and her partner feel part of the team. After surgery, when she had postoperative pains, this induced more stress being pregnant. Caregivers should be aware that in pregnant patients’ complaints that might be considered as normal after surgery can induce a stressful trigger in patients who are already in a stressful situation. The doubts about the original diagnosis of PHPT were shared including the desire of the treating physicians to discuss her case with other experts. Although providing more insecurity, the patient did feel comfortable that this meant that no other surgery was likely to be needed. After the pregnancy she reported that the physical part of the pregnancy did not give her many discomforts but the uncertainty about the well-being of the baby was very stressful. But overall, she was very satisfied with the care given.

In collaboration with these patients we produced a checklist for women in the same position or with a recent diagnosis of PHPT, which might be of help, see Table 1.

**Table 1:** Questions for women in the childbearing age to ask their doctor when diagnosed with a high calcium level

1.	What is the most likely diagnosis causing this high calcium level?
2.	What tests do I need to make the diagnosis more likely? And are these tests influenced by pregnancy?
3.	How will this affect pregnancy?
	What will be the risks for my unborn child?
	What will be the risks for me?
	Which interventions are possible/necessary before, during and after pregnancy?
4.	What can I do to minimalize complaints during and after the pregnancy?
5.	How can we decrease my risk for preeclampsia?
6.	Which drugs might be used during the pregnancy and what are their side effects?
7.	How can you monitor my child?
8.	How will my disease affect me after delivery?
9.	How will this affect my child directly after delivery?
10.	Can I breastfeed?
11.	Who is in my multidisciplinary team and how can I reach them?
12.	What can be done by my general practitioner?
13.	What would be the risk of getting pregnant and to whom can I speak to for counseling?

In both cases patients and partners experienced the pregnancy as a rollercoaster with lot of uncertainties. Whereas both patients were diagnosed with hypercalcemia before pregnancy, patient 1 was counseled extensively in the pre-pregnancy period and even repeatedly got the advice not to become pregnant while patient 2 was never counseled. Also, in patient 2 the diagnosis was changed from PHPT to FHH, the finding of the marked hyperplasia triggered doubts about the original diagnosis of PHPT. In patient 1, the initial decision for surgery would still be made as her calcium values were rising and she did develop nausea and in FHH hyperplasia is also often described.

#### Physiological changes during pregnancy (Fig. 1a)

During pregnancy and lactation there is increased mobilization of calcium in the mother to meet the fetus’ calcium requirements [5, 6]. This is done by increasing intestinal calcium absorption through increasing 1.25 vitamin D production and PTH-related peptide (PTHrP), which is released from placenta and breasts in response to estradiol, placental lactogen, and prolactin [6, 7]. Despite the overlapping modes of action, PTH and PTHrP are produced by different genes on different chromosomes and the gene encoding PTHrP is more complex than for PTH. PTHrP is undetectable in the circulating blood of non-pregnant women, but is produced in a paracrine/autocrine fashion during pregnancy. Lessons from PTHrP or PTH1R gene knockout mice, which die at birth or in utero, emphasize the critical role of PTHrP for normal life [8, 9]. In addition, nascent PTHrP isoforms are processed by members of the prohormone convertase family to at least three fragments: N-terminal PTHrP [1–36], structurally related to PTH; a mid-region PTHrP (38–94); and a C-terminal PTHrP(107–139) with distinct biological properties and receptors. PTHrP is able to act not only via the classical autocrine/paracrine pathways, but also through a so-called intracrine pathway, which involves the translocation of the protein into the nucleus, where it can directly regulate proliferation and apoptosis [10, 11]. PTHrP has multiple activities including control of fetal development, trans-epithelial calcium transfer, lactation, smooth muscle relaxation, and cell growth. The mid-molecular region of PTHrP stimulates placental calcium transport in the fetus, while the COOH terminal region may also affect osteoclast activity. Thus, although PTHrP was discovered as a tumor-derived hypercalcemic factor, its primary physiological role is as a local regulator of many physiological processes especially in early life [12, 13].

**Fig. 1:**
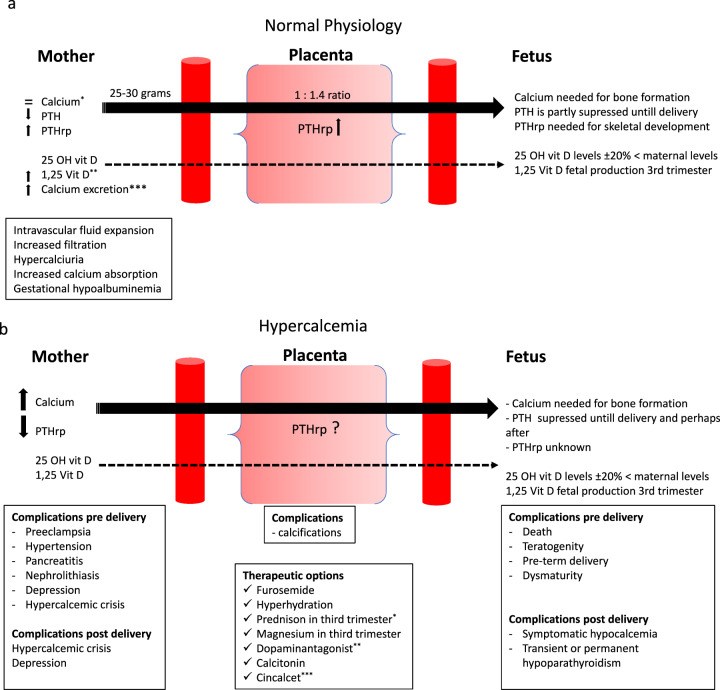
**a** ^*^Intravascular fluid expansion causes gestational hypoalbuminemia. The dilutional hypoalbuminemia of pregnancy leads to a decline in total calcium concentration. Ionized calcium on the other hand, remains stable and may provide more accurate information. ^**^ The increase in 1.25 vitamin D production leads to an increase in intestinal calcium absorption. ^***^“Absorptive hypercalciuria of pregnancy”. Postprandial and 24-h urinary calcium excretion increase after 12 weeks, due to increased intestinal calcium absorption supposedly unrelated to increased formation of 1.25 vitamin D in early pregnancy. Fasting urine samples will not be affected by this phenomenon [45]. **b** ^*^Prednison to reduce 1.25OH formation and thus intestinal calcium absorption. ^**^ Reducing PTH-rp production mammae. ^***^ No clinical data during pregnancy, only case reports. In animal studies no abnormalities observed, but it does pass the placenta [31, 46]. Breastfeeding: in animal studies (rat), cinacalcet is found in milk [46].

#### Maternal causes and consequences of hypercalcemia (Fig. 1b)

Hypercalcemia due to hyperparathyroidism is the most described cause of hypercalcemia during pregnancy. As most reviews focus on maternal and fetal outcomes in PHPT we will not cover this topic in the current paper [14, 15]. Hyperparathyroidism due to diffusely spread hyperthyroid nodules, parathyromatosis, post-parathyroidectomy, in the context of malignancy, MEN syndromes, or in chronic renal disease has been rarely described [15, 16]. Literature data on parathyromatosis is limited to around 20 cases published so far with only one report on a woman diagnosed and treated during pregnancy [17, 18].

FHH is a rare autosomal dominant disorder, with a prevalence of ~1:78.000 though this might likely be higher, as many cases remain asymptomatic lifelong and no causal treatment is available [19–21]. According to the genetic defect causing the pathology, three types of FHH have been defined [21–24]. FHH1 is the most common type and is found in about 65% of all FHH cases [21]. Patients with FHH present with mild to moderate hypercalcaemia, low urinary excretion, and PTH serum levels that are either normal or slightly elevated. Many patients with FHH are misdiagnosed as suffering from PHPT as in the case of our patient. Data on FHH manifested during pregnancy, literature data are sparse. Only four cases were published so far. Walker et al. reported the case of a pregnant patient, initially diagnosed with PHPT, who showed no improvement after PTX. The newborn presented with asymptomatic hypercalcemia; the genetic analysis confirmed the diagnosis of FHH [25]. The newborn presented elevated serum calcium levels with no clinical symptoms, while the genetic analysis confirmed the diagnosis of FHH. Others reported hypercalcemic profiles for which they underwent surgery during pregnancy [26, 27].

PTHrP-induced hypercalcemia during pregnancy or lactation has also been reported, either with or without gigantomastia [28–33]. In the reported cases, associating gigantomastia and increased PTHrP serum values, hypercalcaemia was diagnosed during gestation and resolved only after mastectomy, bromocriptin administration, or termination of pregnancy [29, 31, 32].

After delivery, there is a risk of severe hypercalcemia in women with PHPT who are lactating as PTH-rp production will increase [6, 34]. Especially women with gestational hypercalcemia with gigantomastia should be advised against breastfeeding as this will keep PTHrp levels high. The rise in PTHrp during pregnancy was not seen in patient 1 in whom PTHrp production was fully suppressed during entire pregnancy. The observed rise in calcium levels in patient 2 might in retrospect be due to her different setpoint and PTHrp production. Mental consequences of hypercalcemia are well known and include depression and anxiety [35, 36]. During pregnancy mothers might have additional stress which might be exaggerated by the hypercalcemia.

#### Fetal consequences of hypercalcemia

In the fetus, maternal PHPT can result in fetal growth restriction, severe neonatal hypocalcemia, tetany, and death [36]. In maternal FHH potential fetal complications include mild hypercalcemia, severe hypocalcemia, or neonatal severe hyperparathyroidism, depending on the genotype of the fetus. Because FHH is an autosomal dominant condition, three genetic situations might be encountered by the newborns of FHH mothers: the unaffected and the heterozygous neonates might present with hypocalcaemia induced by the in utero suppression of the parathyroid, the latter being at risk of developing hypercalcemia with hypocalcaemia in the first year of life. The homozygous form of FHH results in a severe neonatal hyperparathyroidism, which requires the surgical removal of the glands during the neonatal phase [37, 38].

Most outcomes on the fetal consequences of hypercalcemia were described in reports on patients with PHPT. Untreated hypercalcemia was associated with fetal or neonatal complications in the majority of cases [3, 36, 39, 40]. However, these findings are based on early literature of PHPT patients when hypercalcemia was often diagnosed in an advanced stage and more recent literature has shown that, compared to age-matched controls, women with PHPT had no difference in most pregnancy outcomes, including stillbirths [39]. That same study also showed that Apgar scores and anthropometric measurements were similar to babies born in uncomplicated pregnancies. However, this recent study did not report on maternal outcomes of preeclampsia. Other recent studies also report lower complications rates and good treatment outcomes when the hypercalcemia is moderate and not severe [1, 41].

Elevated maternal serum calcium leads to hypercalcemia in the fetus, which causes fetal parathyroid gland suppression. After delivery, these glands are still suppressed causing fetal hypocalcemia, which is usually transient (lasting 3–5 months after birth), but permanent hypoparathyroidism has anecdotally been described [42].

In the case of parathyromatosis diagnosed during pregnancy, Edling et al. reported that the infant presented initially with high calcium and low PTH levels, which normalized rapidly, with no further complications [18]. Newborns of mothers presenting with humoral hypercalcemia of pregnancy were having normal calcium levels at birth [29, 30, 33].

Diagnosing hypercalcemia during pregnancy might be challenging due to both physiological changes in calcium homeostasis, as well as due to the underlying cause for the hypercalcemia; changes in urinary calcium excretion vary during daytime, introducing difficulty in using urinary calcium indices as a diagnostic tool. In non-pregnant population, the measurement of calcium/creatinine clearance ratio can be useful in distinguishing between PHPT(>0.02 mmol/mmol) and FHH(<0.01 mmol/mmol). However, it is known that almost 20% of FHH patients might present with a calcium/creatinine ratio >0.01, and individuals with PPHT and simultaneous vitamin D deficiency might show lower than expected calcium excretion [43]. Genetic testing, when available, should be considered for confirming the clinical suspicion of FHH. More than 130 CaSR mutations have been reported so far in relation to FHH1, fact that explains the phenotypical variations in relation to the observed differences in serum calcium and PTH levels [21, 44].

During pregnancy, the distinction between FHH and PHPT is further complicated by the physiological changes in maternal calcium metabolism: increased breast and placental production of PTHrP, that together with the placental lactogen, leads to an increase in 1,25-(OH)2- vitamin D and a reduction of the PTH levels. As a result, the intestinal calcium absorption and the urine calcium excretion are physiologically increased [5, 45]. Treatment is depending on the underlying cause and might be invasive or conservative, but all decisions need good counseling, as it concerns not only the mother, but also the fetus and partner as well. Also we, as physicians, need to be aware that the treatment does not stop with the deliverance of a healthy baby and that the postpartum period might actually be the period with the highest risk for the mother due to the risk of sudden rises in calcium level and to excessive mental stress caused by possible events during pregnancy or delivery.

But foremost, irrespective of the underlying cause of hypercalcemia, patients and partners need to be closely involved in the decision processes in order to ensure overall good outcomes for mother and child.
